# Reversible Adsorption of Polycarboxylates on Silica Fume in High pH, High Ionic Strength Environments for Control of Concrete Fluidity

**DOI:** 10.1021/acs.langmuir.1c02419

**Published:** 2022-01-28

**Authors:** Brant Walkley, Daniel A. Geddes, Taku Matsuda, John L. Provis

**Affiliations:** † Department of Materials Science and Engineering, The University of Sheffield, Sheffield S1 3JD, UK; ‡ Department of Chemical and Biological Engineering, The University of Sheffield, Sheffield S1 3JD, UK; § Construction Material Group and Geotechnical Technology Department, Sumitomo Mitsui Construction Co., Ltd., Nagareyama-Shi, Chiba 270-0132, Japan

## Abstract

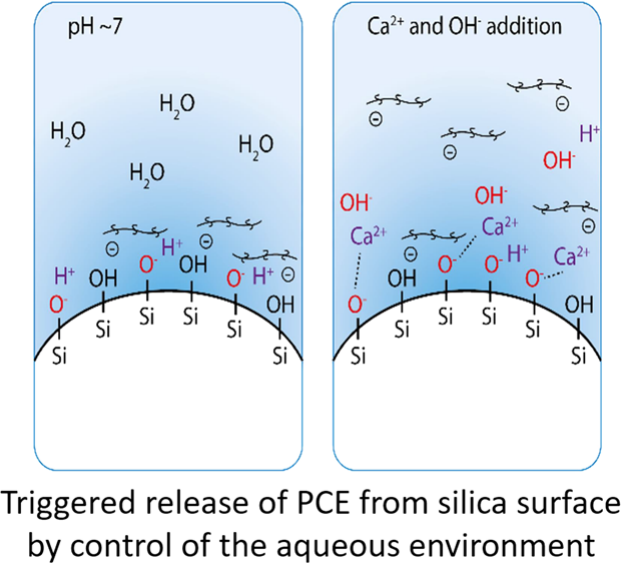

Polycarboxylate-based superplasticizers are essential for production of ultrahigh-performance concrete (UHPC), facilitating particle dispersion through electrostatic repulsion and steric hindrance. This study examines for the first time the effect of changes in pH, ionic strength, and charge on the adsorption/desorption behavior of a polycarboxylate-based superplasticizer on silica fume in aqueous chemistries common in low-CO_2_ UHPC. Data from total organic carbon measurements, Fourier transform infrared and nuclear magnetic resonance spectroscopy, and zeta potential measurements reveal the silica surface chemistry and electrokinetic properties in simulated UHPC. Addition of divalent cations (Ca^2+^) results in polycarboxylate adsorption on silica fume via (i) adsorption of Ca^2+^ ions on the silica surface and a negative zeta potential of lower magnitude on the silica surface and (ii) reduction of polycarboxylate anionic charge density due to complexation with Ca^2+^ ions and counter-ion condensation. Addition of OH^–^ ions results in polycarboxylate desorption via deprotonation of silanol groups and a negative zeta potential of greater magnitude on the silica surface. Simultaneous addition of both Ca^2+^ and OH^–^ results in rapid polycarboxylate desorption via (i) formation of an electric double layer and negative zeta potential on the silica surface and (ii) an increase in polycarboxylate anionic charge density due to deprotonation of the carboxylate groups in the polymer backbone, complexation with Ca^2+^ ions, and counter-ion condensation. This provides an explanation for the remarkable fluidizing effect observed upon addition of small amounts (1.0 wt %) of a solid, powdered Ca source to fresh, low-CO_2_, UHPC, which exhibits significantly higher fresh state pH (>13) than those based on Portland cement (pH 11).

## 1 Introduction

Ultrahigh-performance concrete (UHPC) is produced with a low water/cement (w/c) ratio, which provides the low porosity required for outstanding strength and durability. Reducing the w/c ratio has a negative effect on the workability, and the inclusion of a superplasticizer within the fresh mixture allows for improved workability and flow.^1,2^ Modern UHPCs comprise significant quantities of supplementary cementitious materials (SCM) such as silica fume, blast furnace slag, and coal fly ash, which enhance and control physical properties, e.g., strength, durability, and reduce-associated CO_2_ emissions.^3^ To produce UHPC with satisfactory workability and flow characteristics, a detailed understanding of the interactions between an organic superplasticizer and inorganic cement and SCM particles is essential.

Silica fume is a reactive, amorphous form of SiO_2_ produced as a byproduct of semiconductor Si manufacture, comprising spherical particles with an extremely small (tens of nm) particle size.^4^ It is commonly blended with Portland cement at relatively low levels (up to 10 wt %) to enhance performance, improving paste cohesion and rheological properties in the fresh state while promoting strength development and durability in the hardened state via pozzolanic reactivity.^5^ Silica fume has a very high surface area of between 15 and 30 m^2^/g due to its small particle size.^6^ Within modern blended Portland cement formulations, with typical specific surface areas of between 0.3 and 0.5 m^2^/g,^6^ even this modest dose of silica fume can account for as much as 85% of the particle surface area in the cementitious powders. Consequently, inclusion of silica fume within blended cement formulations dramatically increases the w/c ratio required to achieve suitable flowability. Superplasticizing dispersants are therefore required to achieve satisfactory flow at the low w/c ratios that are needed to achieve the low porosity and high strength and durability in UHPC.

Polycarboxylate-based superplasticizers have been shown to be the most effective dispersants for cementitious formulations containing silica fume.^7^ Polycarboxylate-based superplasticizers interact with cement and silica fume particles via surface adsorption through hydrogen bonding between terminal hydroxyl groups on polyethylene glycol graft chains and silanol groups on the silica surface.^8^ This facilitates particle dispersion through both electrostatic repulsion and steric hindrance.^9−12^

**Figure 1:**
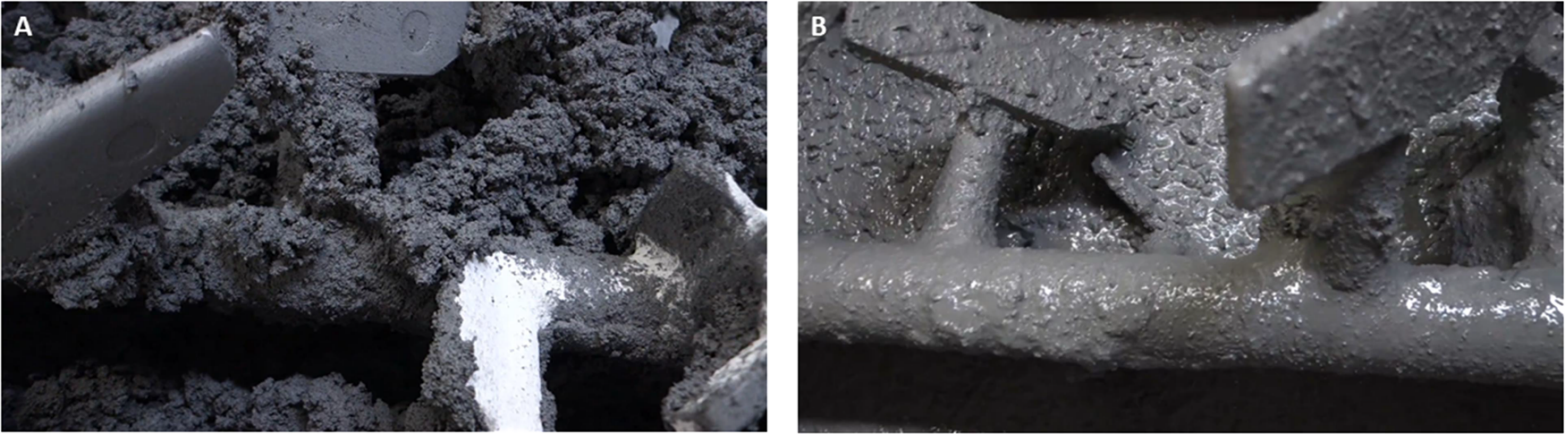
Images taken from the video (Video S1), showing the effect of addition of the powdered Ca source (primarily CaO and other calcium salts, sourced commercially as an “expansive additive”) on a high-strength alkaline-earth-activated concrete produced from silica fume, fly ash, and blast furnace slag, without inclusion of Portland cement, and with a very low water/cement ratio (w/c = 0.16).^13^ Panel (A) represents the state before addition of the powdered Ca source (where the paste is not fluid at all), and panel (B) represents the state approximately 1 min after addition of the powdered Ca source (where the paste is very fluid).

Novel, high-strength alkaline-earth-activated concretes with a very low w/c ratio of 0.16 and a very low embodied CO_2_ have recently been produced from silica fume, fly ash, and blast furnace slag, without inclusion of Portland cement.^13^ The cements used in these concretes comprise a minimum of 96 wt % (dry basis) industrial byproducts and yield concretes with very high performance (strength and durability) at an exceptionally low water content, with compressive strengths of 90 and 150 MPa after 28 days and 2 years of curing, respectively.^13^ These concretes initially exhibit very low fluidity and flowability (Figure 1A), despite containing a high dose of polycarboxylate superplasticizer intended to give high flowability at such a low water content. However, a remarkable fluidizing effect is observed upon addition of a very small amount (1.0 wt %) of a solid, powdered Ca source during mixing^13,14^ (primarily calcium oxide and other calcium salts, obtained in that study via a commercially available “expansive additive”) (Figure 1B). The addition of this powdered Ca source (containing calcium oxide and other calcium salts) also instigates the pozzolanic/latent hydraulic reaction of the inorganic SCM particles.^14^ We hypothesize that the superplasticizer is initially sorbed onto the very high surface area of the silica fume particles, and then, the increase in pH induced by the addition of Ca^2+^ provided by the soluble calcium oxide causes rapid desorption of the superplasticizer from the silica fume particles, with a dramatic fluidizing effect. Such a mechanism has not previously been documented in the discussion of this type of high-performance concrete and could provide a new pathway to the production of durable, low-CO_2_ concretes, which are readily usable at a very low water content.

However, due to significant differences between the aqueous and solid state chemistry of Portland cement and alkali/alkaline-earth-activated cement systems, the behavior of dispersants in each case differs significantly, so the hypothesis described in the preceding paragraph requires fundamental examination to be validated. In particular, the reversibility of adsorption of superplasticizing dispersants on high surface area silica particles, in the high pH and high ionic strength environments common in fresh alkali/alkaline-earth-activated cement pastes, remains poorly understood.

While many studies have examined adsorption phenomena of polycarboxylate-based superplasticizers in Portland cement and Portland cement blended with silica fume,^6,11,12,15−17^ few have examined this in alkali-activated cement systems, and to our knowledge, none have done so for polycarboxylate-based superplasticizers in novel alkaline-earth-activated cement systems. It is therefore crucial to understand the surface chemistry and adsorption phenomena of polycarboxylate-based superplasticizers on silica surfaces in conditions simulating the aqueous phase in fresh state alkaline-earth-activated cements.

In this study, we examine the effect of changes in pH, ionic strength, and charge on the reversibility of adsorption of a polycarboxylate-based superplasticizer on silica fume. Through quantification of data from total organic carbon measurements, Fourier transform infrared spectroscopy, solution state ^1^H, ^13^C, and ^29^Si nuclear magnetic resonance (NMR) spectroscopy and solid state ^29^Si MAS magic angle spinning (MAS) NMR, and zeta potential measurements, we reveal the silica surface chemistry and electrokinetic properties in these simulated pore solutions. We also propose a model for the mechanism by which the aqueous chemistry controls the adsorption/desorption phenomena of these organic molecules, which causes the dramatic fluidization observed in Figure 1 during mixing of fresh alkaline-earth-activated cement pastes.

## 2 Materials and Methods

### 2.1 Overview of Methodology

The adsorbed amounts of a polycarboxylate-based superplasticizer on silica fume were measured using the depletion method based on the principle that the interaction between the polycarboxylate-based superplasticizer and silica fume is due only to surface adsorption. This hypothesis was validated by solution state ^29^Si NMR and solid state ^29^Si MAS NMR measurements of silica fume before and after adsorption (details discussed in Section 3.1.3). The amount of non-adsorbed superplasticizer remaining in solution after each adsorption experiment was determined by analyzing the total organic carbon (TOC) content of the solution. This was independently confirmed by quantification of solution state ^1^H and ^13^C NMR measurements for each colloidal dispersion and by Fourier transform infrared spectroscopy measurements for the solids before and after adsorption. The mechanism of sorption/desorption of the polycarboxylate-based superplasticizer on silica fume was determined using zeta potential measurements of each colloidal dispersion. Details of all characterization experiments are provided below.

### 2.2 Sample Preparation

**Figure 2:**
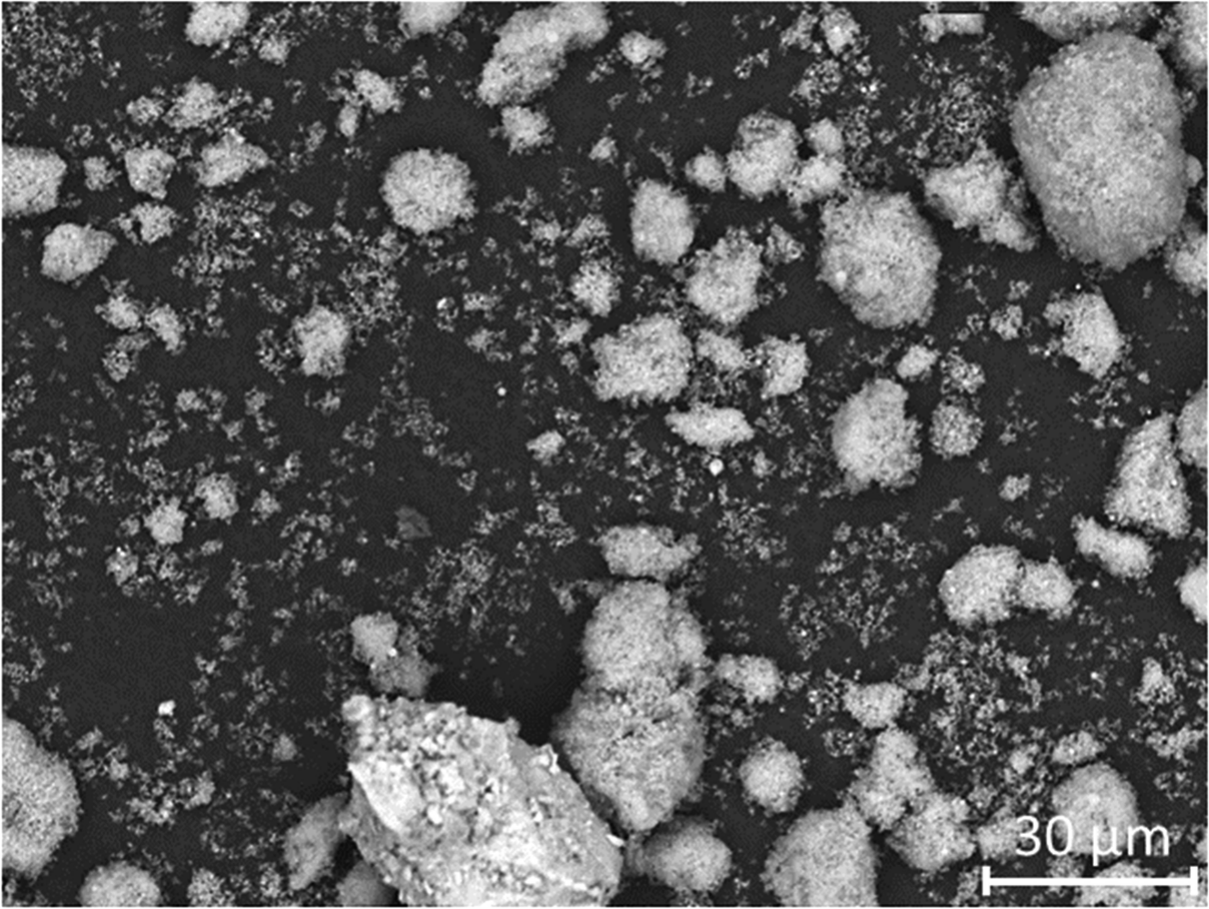
Scanning electron microscopy (secondary electron) image, collected at 1000× magnification, showing the particulate and agglomerated nature of the silica fume.

The silica fume used in this study was obtained from Efaco, Japan, and exhibited a chemical composition, particle size distribution, and morphology, as shown in Table 1 and Figure 2, respectively. The un-agglomerated silica fume particles exhibit diameters on the order of tens of nm (smallest particles visible in Figure 2), although laser particle size distribution analysis data (not shown) identify a large number of particles with apparent diameters on the order of tens of μm and a modal particle size of 40 μm due to particle agglomeration.

**Table 1:** Chemical Composition and Loss on Ignition at 1000 °C of Silica Fume

component	oxide, mass % by XRF
SiO_2_	93.98
Al_2_O_3_	0.54
Fe_2_O_3_	1.52
CaO	0.31
MgO	0.52
Na_2_O	0.36
K_2_O	0.61
loss on ignition	2.16

Colloidal dispersions containing 5 wt % silica were produced by addition of silica fume to solutions of 15, 9, and 6 g/L polycarboxylate-based superplasticizers (Sikament 1200 N, Sika Ltd., Japan; Mw = 18 kDa, Mn = 12 kDa, Mw/Mn = 1.5) in milliQ water. Control solutions comprising 15, 9, and 6 g/L superplasticizers in milliQ water (positive control) and milliQ water with no addition of superplasticizer (negative control) were produced for comparison. As expected, the data for negative control samples showed negligible TOC (within the error of the measurement) and were omitted from subsequent figures for clarity. Further information about the polycarboxylate chain structure of the superplasticizer used in this work, or any ancillary compounds in the formulated superplasticizer product, is not available due to confidentiality restrictions in the use of this commercial product. However, as the same superplasticizer is used for all tests, the amount of superplasticizer remaining in solution after filtration can be quantified relative to the control samples.

The effect of aqueous chemistry on sorption/desorption phenomena within these dispersions was investigated by separately raising the pH to 12.3 by addition of NaOH (Sigma Aldrich) to a concentration of 20 mmol/L and by addition of Ca^2+^ (as Ca(NO_3_)_2_, Sigma Aldrich) to a concentration of 440 mmol/L (chosen to achieve a Ca/Si molar ratio equivalent to typical values in fresh low-CO_2_ alkaline-earth-activated concrete^13,14^). This was done to determine whether the dispersion of the solid particles and rapid fluidization of the fresh cement paste were a result of (i) the high pH environment or (ii) the change in ionic strength induced by the addition of divalent cations provided by dissolution of the CaO-rich additive in the fresh cementitious mixture.

To quantify the effect of addition of hydroxyl ions on the sorption/desorption phenomena in these colloidal dispersions, NaOH was added to a concentration of 440 mmol per gram of polycarboxylate (i.e., holding the [OH^–^]/mass polycarboxylate ratio constant) in separate colloidal dispersions containing 5 wt % silica in solutions of 15, 9, and 6 g/L polycarboxylate-based superplasticizers in milliQ water. This results in solution pH values of 13.5, 13.3, and 13.1 for samples containing 15, 9, and 6 g/L superplasticizers in milliQ water, respectively, and allows quantification of the effect of addition of hydroxyl ions per gram of superplasticizer. After addition of all constituents, each colloidal dispersion was left to stand for 1 min prior to separation of solid and aqueous components using a polymer vacuum filter (pore size 0.22 μm). Both the solid and aqueous components were collected and stored in sealed containers prior to analysis.

### 2.3 Characterization

#### 2.3.1 Scanning Electron Microscopy

Scanning electron microscopy (SEM) analysis was performed using a Hitachi TM3030 using a sample that is coated in carbon to prevent charging, an accelerating voltage of 15 kV, and a working distance of approximately 8 mm.

#### 2.3.2 Total Organic Carbon Measurements

Total organic carbon (TOC) data were acquired by combustion over an oxidation catalyst at 680 °C using a Shimadzu total organic carbon V_CPH_/_CPN_ analyzer, with 150 mL/min zero grade air as the carrier gas. The amount of polycarboxylate superplasticizer adsorbed on particles was calculated from the difference between the TOC content of the control solutions and the respective colloidal dispersions.

#### 2.3.3 Nuclear Magnetic Resonance Spectroscopy

Solution state NMR data were acquired using a Bruker AVANCE III 400 spectrometer, using an observe configuration (^1^H on outer coil) multinuclear two-channel probe, yielding a Larmor frequency of 400.2 MHz for ^1^H, 100.58 MHz for ^13^C, and 79.47 MHz for ^29^Si. Sample suspensions in D_2_O containing 0.65 M dimethyl sulfoxide-d6 (DMSO) as the internal standard were brought to a total volume of 0.6 mL and loaded into 5 mm NMR tubes. Quantitative ^1^H NMR spectra (using the DMSO internal standard) were acquired using a π/2 pulse (Bruker sequence: zg), using 64 k acquisition points over an 8.2 kHz acquisition window using 16 transients and a relaxation delay of 120 s. This relaxation delay was chosen as sufficient for complete relaxation following a T1 determination of the components in the solution. Solvent (water)-suppressed spectra were acquired using a NOESY presaturation sequence (Bruker sequence: noesygppr1d), using 64 k acquisition points over an 8.2 kHz acquisition window using 128 transients, a mixing time of 10 ms, and a relaxation delay of 2 s. ^13^C spectra were acquired in a pseudo-quantitative way using the uniform driven equilibrium Fourier transform (UDEFT) technique to minimize relaxation delay and NOE (Bruker sequence: udeft), using 21.4 k acquisition points over a 23.8 kHz acquisition window, using 1600 transients. ^29^Si spectra were acquired using inverse gated decoupling and a π/6 flip angle (Bruker sequence: zgig30), using 64 k acquisition points over a 32.1 kHz acquisition window using 344–1024 transients and a relaxation delay of 10 s.

The solid state ^29^Si MAS NMR spectrum of anhydrous silica fume was acquired on a Bruker Avance III HD 500 spectrometer at 11.7 T (B0) using a 4.0 mm dual resonance CP/MAS probe, yielding a Larmor frequency of 99.35 MHz. ^29^Si MAS NMR data were acquired using a 4 μs non-selective (π/2) excitation pulse, a 15 s relaxation delay, a total of 512 transients, and spinning at 12.5 kHz.

#### 2.3.4 Fourier Transform Infrared Spectroscopy

Samples for FTIR spectroscopy analysis were prepared by mixing 2 mg of the sample with 200 mg of KBr and pressing the mixture into a pellet. FTIR spectra were measured using a Perkin Elmer Frontier Mid FT-IR spectrometer equipped with a deuterated triglycine sulfate (DTGS) detector and KBr beam splitter optical system, scanning 16 times at a resolution of 4 cm^–1^.

#### 2.3.5 Zeta Potential Measurements

Zeta potential measurements were performed using a Malvern Instruments Zetasizer Nano series instrument. Zeta potentials were determined via the Henry equation using the Smoluchowski approximation.^18^

## 3 Results and Discussion

UHPC formulations have a very low water content to provide low porosity required for very high strength. The low water content, however, reduces the workability and flow characteristics of fresh UHPC. It is therefore essential that UHPC mixtures are formulated correctly to achieve the desired workability, flow, and water content. This is typically achieved by inclusion of a superplasticizer within the fresh mixture that allows for improved workability and flow.^1,2^

In recently reported novel, high-strength alkaline-earth-activated concretes produced from silica fume, fly ash, and blast furnace slag, without inclusion of Portland cement,^13^ the desired workability, flow, and water content (w/c = 0.16) were achieved by preparation of the fresh mixture containing a polycarboxylate-based superplasticizer and subsequent addition of a solid, powdered Ca source (during mixing^13,14^ (primarily calcium oxide and other calcium salts, obtained in that study via a commercially available “expansive additive”). These concretes initially exhibited very low fluidity and flowability (Figure 1A), despite containing a high dose of polycarboxylate superplasticizer intended to give high flowability at such a low water content. After addition of a very small amount (1.0 wt %) of the solid, a powdered Ca source during mixing^13,14^ a remarkable fluidizing effect is observed (Figure 1B).

The work in the study presented here reveals the silica surface chemistry and electrokinetic properties in chemically simplified environments that represent the various states observed in fresh alkaline-earth-activated cement pastes, as discussed above. In particular, we have replicated the order of addition of constituents (i.e., silica fume, superplasticizer, Ca^2+^ ions, and OH^–^ ions) to provide an understanding of the effect of each constituent on adsorption/desorption of the superplasticizer during the initial stages of mixing. This reveals the mechanism by which the aqueous chemistry controls the adsorption/desorption phenomena of the polycarboxylate-based superplasticizer, which causes the dramatic fluidization observed in Figure 1 during mixing of fresh alkaline-earth-activated cement pastes through the control of pH and ionic strength.

### 3.1 Effect of pH and Ionic Strength on Adsorption/Desorption of Polycarboxylate on Silica Fume

#### 3.1.1 Total Organic Carbon (TOC)

**Figure 3:**
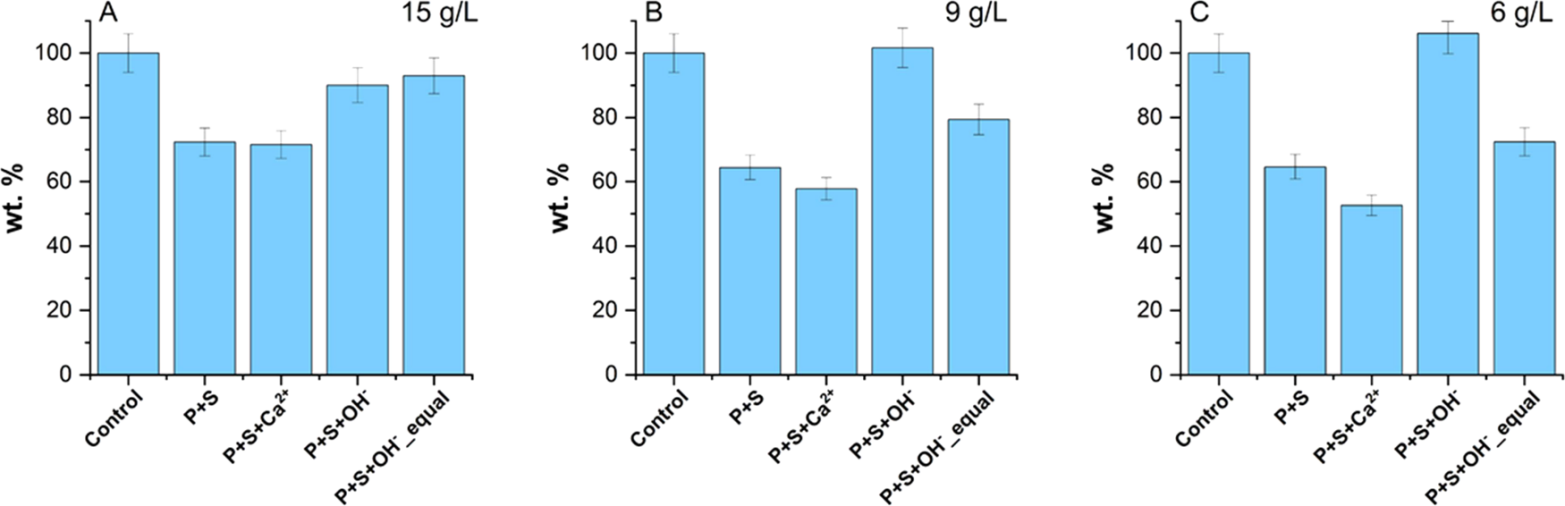
Mass of total organic carbon remaining in the polycarboxylate solution (relative to the respective control samples) after addition of 5 wt % silica fume (P + S), addition of 5 wt % silica fume and adjustment of ionic strength by addition of Ca^2+^ (P + S + Ca^2+^), and addition of 5 wt % silica fume and adjustment of pH by addition of OH^–^ (P + S + OH^–^). Data are shown for polycarboxylate concentrations of (A) 15 g/L, (B) 9 g/L, and (C) 6 g/L as marked. Data obtained from total organic carbon content measurements.

The mass of total organic carbon remaining in the polycarboxylate solution (relative to the control samples) after sorption onto silica fume under different aqueous solution environments is shown in Figure 3. Without adjustment of the aqueous chemistry (i.e., mimicking the case in which no CaO-rich additive is added to the concrete mixture) between 28 and 36 wt % of the superplasticizer was adsorbed to the surface of silica fume. This will reduce the effectiveness of the polycarboxylate in dispersing solid particles in the fresh concrete mixture and corresponds to the state shown in Figure 1A where the paste is not fluid at all. The solid and aqueous components were separated using a polymer vacuum filter (pore size 0.22 μm); however it is possible that very small solid particles remained in solution after filtration. These very small particles will have a larger surface area, relative to the larger particles that will have been removed, which may explain the relatively modest drop in TOC upon sorption compared to the very dramatic changes in fluidity discussed above.

At high superplasticizer concentrations (15 g/L; Figure 3A), addition of Ca^2+^ as Ca(NO_3_)_2_ does not result in an increase in adsorption compared with the control dispersions comprising only the superplasticizer and silica fume. This contrasts with Figure 3B,C, where addition of Ca^2+^ as Ca(NO_3_)_2_ results in a further increase in adsorption (compared with the control dispersions comprising only the superplasticizer and silica fume, in the absence of any Ca^2+^ additive). The magnitude of this increased adsorption is greater at lower superplasticizer concentrations (e.g., at 6 g/L c.f. 9 g/L). This indicates that silica fume surface sites for adsorption may be saturated at higher superplasticizer concentrations. Conversely, addition of OH^–^ to raise the pH to 12.3 results in rapid desorption of the superplasticizer from silica fume, with complete desorption observed for superplasticizer concentrations of 6 and 9 g/L (Figure 3B,C). This shows that it is the high pH environment provided by dissolution of the CaO-rich additive in the fresh cementitious mixture, rather than just the change in ionic strength induced by the addition of divalent cations, that enables dispersion of the solid particles and rapid fluidization of the paste.

#### 3.1.2 Fourier Transform Infrared (FTIR) Spectroscopy

**Figure 4:**
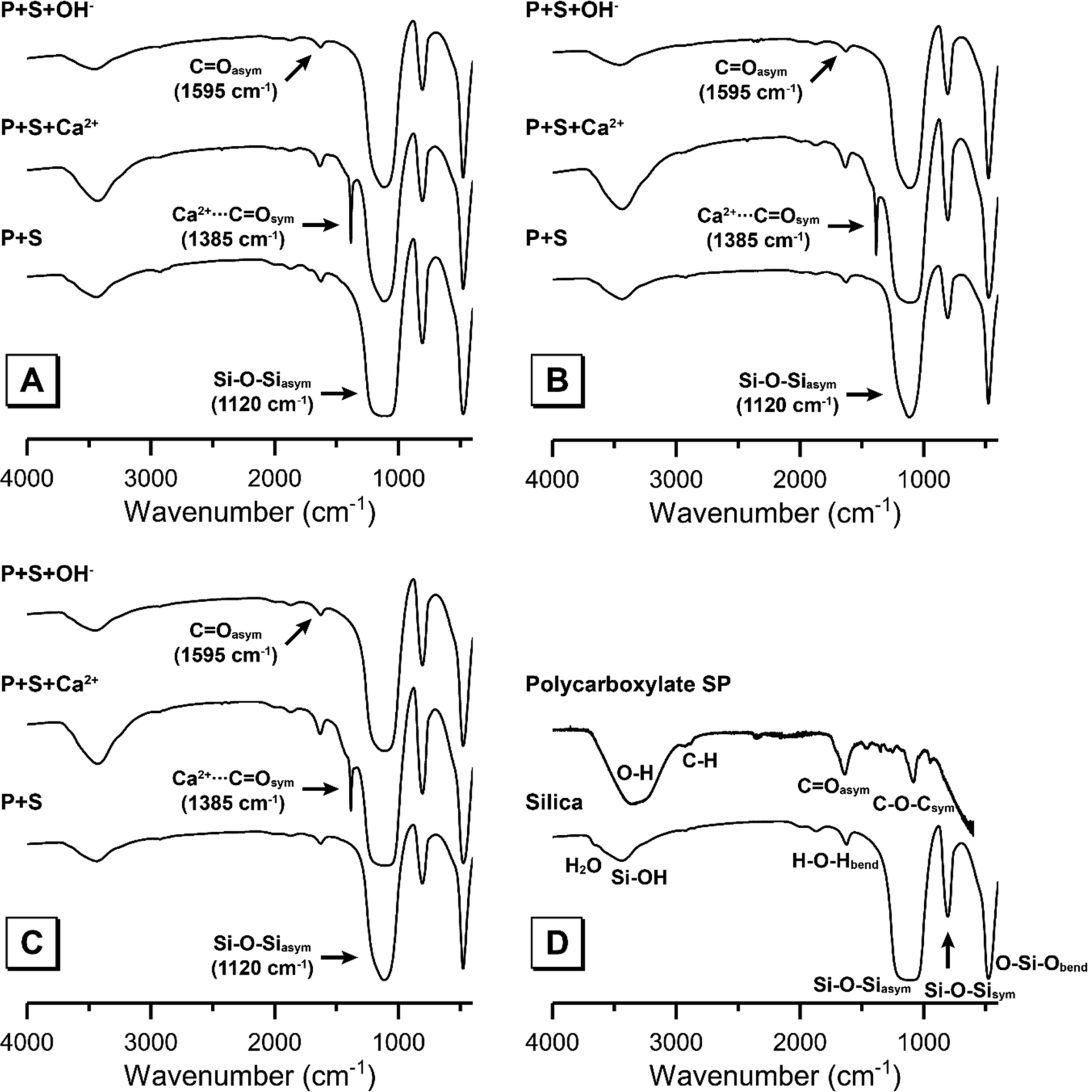
FTIR spectra of the solid phase (silica fume plus any adsorbed polycarboxylate) remaining after filtration of silica fume suspensions after addition of 5 wt % silica fume (P + S), addition of 5 wt % silica fume and adjustment of ionic strength by addition of Ca^2+^ (P + S + Ca^2+^), and addition of 5 wt % silica fume and adjustment of pH by addition of OH^–^ (P + S + OH^–^), as marked. Data are shown for polycarboxylate concentrations of (A) 15 g/L, (B) 9 g/L, and (C) 6 g/L as marked and (D) untreated silica fume and the polycarboxylate superplasticizer.

FTIR data can be used to confirm the presence of polycarboxylate adsorbed on the surface of silica. The FTIR spectrum for silica fume (Figure 4) exhibits a high intensity band spanning from approximately 1020 to 1220 cm^–1^ that is assigned to asymmetric stretching of Si–O–Si bonds,^19^ a lower intensity band at 805 cm^–1^ that is assigned to symmetric stretching of Si–O–Si bonds,^19^ a high intensity band at 480 cm^–1^ assigned to O–Si–O bending (deformation) vibrations,^20^ a band at 3400 cm^–1^ due to stretching vibrations of O–H bonds in H_2_O physisorbed to the surface of silica fume, and a band at 3700 cm^–1^ due to stretching of Si–O–H linkages.^20^

The FTIR spectrum of the polycarboxylate-based superplasticizer (Figure 4) exhibits a band at 1140 cm^–1^ that is assigned to asymmetric stretching vibrations of C–O–C linkages, bands at 1415 and 1595 cm^–1^ that are assigned to symmetric and asymmetric stretching vibrations of C=O bonds, respectively, a band at 2900 cm^–1^ assigned to C–H stretching vibrations, and a band at 3400 cm^–1^ due to stretching vibrations of hydroxyl groups.^15^

The FTIR spectra of the solid phases separated from each colloidal dispersion after adsorption, modification of aqueous chemistry, and filtration are shown in Figure 4. All spectra are dominated by bands attributed to the aforementioned sites in silica fume and the superplasticizer. An additional band at 1385 cm^–1^ is observed in all solid phases from filtered colloidal dispersions containing Ca^2+^ ions, resulting from symmetric stretching vibrations of C=O bonds within Ca^2+^-carboxylate complexes.^21^

Comparing the spectra for each sample, the bands due to stretching vibrations of hydroxyl groups,^15^ asymmetric stretching vibrations of C=O bonds in carboxylate groups (including those complexed with Ca^2+^), and symmetric stretching vibrations of C=O bonds in carboxylate groups within the superplasticizer exhibit higher intensities in the spectra for solid phases from filtered colloidal dispersions containing Ca^2+^ ions. The intensities of these bands are similar in spectra for the solid phases from colloidal dispersions without adjustment of the aqueous chemistry and with addition of OH^–^ ions.

These trends indicate greater adsorption of the polycarboxylate-based superplasticizer to the silica surface in solid phases from filtered colloidal dispersions containing Ca^2+^ ions and no increase in adsorption to the silica surface in solid phases from filtered colloidal dispersions with added NaOH when compared to that of colloids without modification of the aqueous chemistry. These observations confirm the TOC results discussed above, where addition of Ca^2+^ to the colloidal dispersions is driving increased adsorption of the polycarboxylate-based superplasticizer, and addition of OH^–^ ions causes desorption of the polycarboxylate-based superplasticizer from the surface of silica.

#### 3.1.3 Nuclear Magnetic Resonance (NMR) Spectroscopy

**Figure 5:**
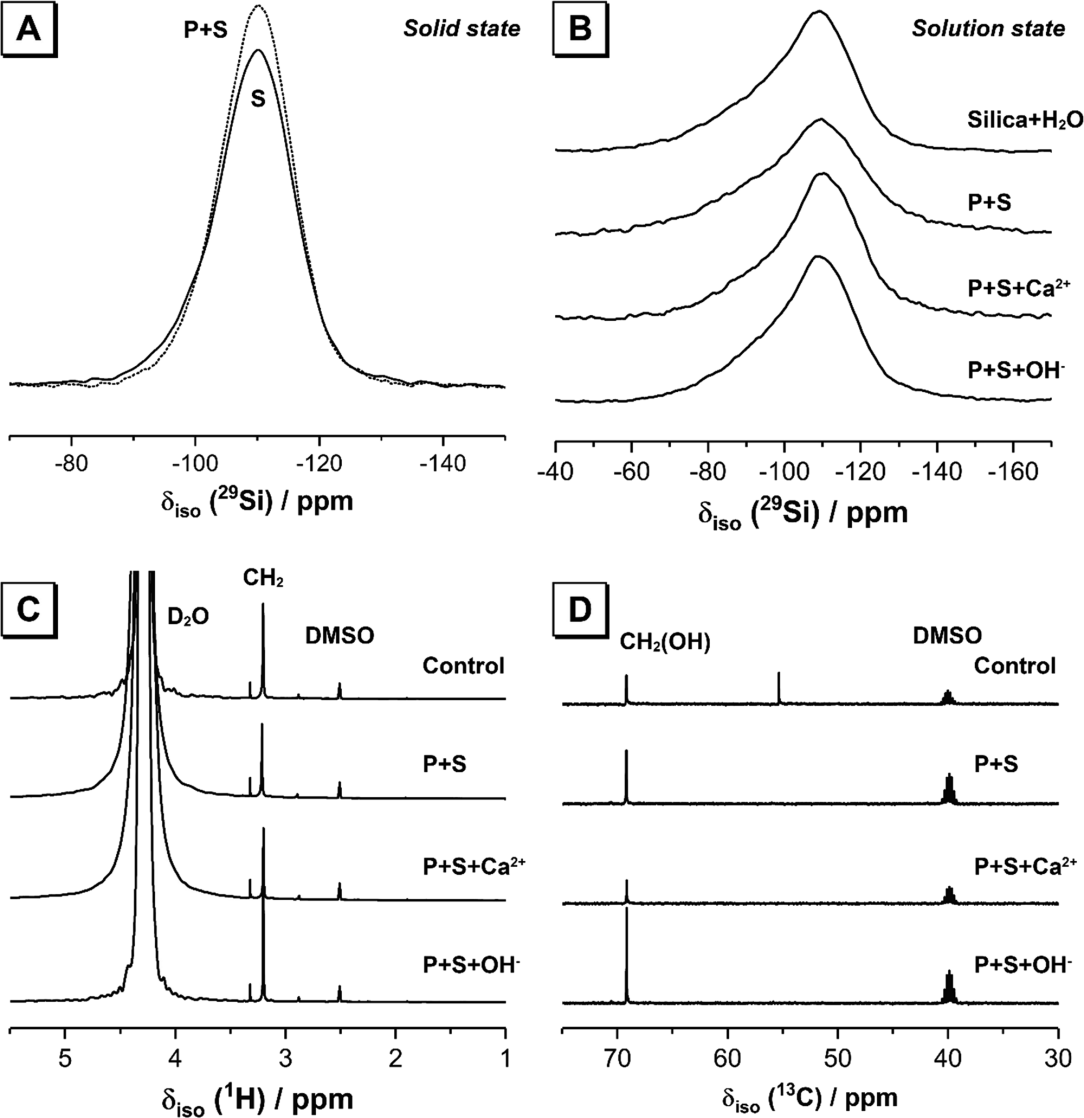
(A) Comparison of solid state ^29^Si MAS NMR data (*B*_0_ = 11.7 T, ν_R_ = 12.5 kHz) for anhydrous silica fume (solid line, labeled S) and the solid phase (dotted line, labeled P + S) obtained after filtration of the colloidal dispersion with a polycarboxylate concentration of 15 g/L. (B) Solution-state ^29^Si NMR data for the colloidal dispersions after addition of 5 wt % silica fume (P + S), addition of 5 wt % silica fume and adjustment of ionic strength by addition of Ca^2+^ (P + S + Ca^2+^), and addition of 5 wt % silica fume and adjustment of pH by addition of OH^–^ (P + S + OH^–^). Solution-state (C) ^1^H and (D) ^13^C NMR data for the aqueous components obtained after filtration of the colloidal dispersions, after addition of 5 wt % silica fume (P + S), addition of 5 wt % silica fume and adjustment of ionic strength by addition of Ca^2+^ (P + S + Ca^2+^), and addition of 5 wt % silica fume and adjustment of pH by addition of OH^–^ (P + S + OH^–^). Data are shown for a polycarboxylate concentration of 15 g/L.

The solid state ^29^Si MAS NMR spectrum of anhydrous silica fume (Figure 5A) exhibits a single broad resonance at δ_iso_ = −110 ppm, indicating that it comprises a distribution of tetrahedral (predominantly Q^4^) Si sites.^22^ The line shape of both the solid state ^29^Si MAS NMR spectrum for the solid phase obtained by filtering the colloidal dispersion (Figure 5A) and the solution state ^29^Si NMR spectra for the colloidal dispersions after adsorption of polycarboxylate in each of the test environments considered (Figure 5B) are almost identical to those of untreated silica fume obtained in each respective experiment. This shows that the test methods applied for adsorption/desorption of the polycarboxylate-based superplasticizer and modification of aqueous chemistry do not alter the structure of silica fume within the timeframe that the experiments were performed. There is no significant formation of surface-bound calcium silicate hydrates during these experiments where calcium was added, and any dissolution induced by the elevated pH of the NaOH addition test did not significantly alter the structure of the remaining solid silica fume particles. This supports the hypothesis that the interaction between the polycarboxylate-based superplasticizer and silica fume in the aqueous environments tested here is due only to surface adsorption and validates the depletion methodology used for the adsorption experiments in this work. The significant line width of the Q^4^ Si species in the solution state NMR is expected for silica fume and other similarly sized (colloidal) silica particles^23^ and shows that minimal or no hydration and dissolution reactions have occurred in the silica fume during testing.^24^

^1^H NMR data for the aqueous components of each sample (Figure 5C) were acquired in the solution state. As discussed above, the aqueous and solid components of each colloidal dispersion were separated using a polymer vacuum filter (pore size of 0.22 μm). It is therefore possible that very small solid particles remained in solution after filtration, to which polycarboxylate may sorb. It is assumed, however, that polycarboxylate sorbed to the surface of these small solid particles will have very low mobility and will therefore be unobservable in the ^1^H NMR data in Figure 5.

Each ^1^H NMR spectrum exhibits a resonance at 3.20 ppm, assigned to hydroxyl group protons in the carboxylic acid terminus in the polycarboxylate-based superplasticizer.^25^ Although detailed information about the polycarboxylate chain length in the superplasticizer used in this work is not available due to confidentiality in the use of this commercial product, as the same superplasticizer is used for all tests, the amount of superplasticizer remaining in solution after filtration can be quantified relative to the control sample. ^13^C NMR data for all colloidal dispersions (Figure 5D) exhibit a resonance at 69.2 ppm, assigned to carbon atoms in −CH_2_(OH) groups in the polycarboxylate backbone in the superplasticizer.^15^

**Figure 6:**
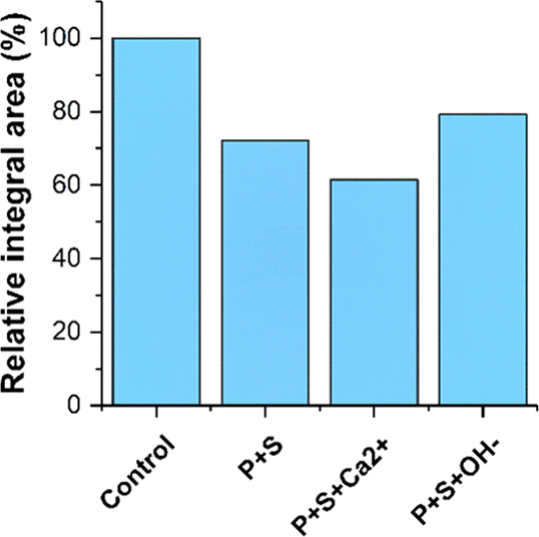
Relative integral area (proportional to amount) of polycarboxylate remaining in solution (relative to the control samples), with a starting concentration of 15 g/L, as determined by ^1^H NMR measured in the solution state. Data are shown for the aqueous component obtained after filtration of colloidal dispersions, after addition of 5 wt % silica fume (P + S), addition of 5 wt % silica fume and adjustment of ionic strength by addition of Ca^2+^ (P + S + Ca^2+^), and addition of 5 wt % silica fume and adjustment of pH by addition of OH^–^ (P + S + OH^–^).

Calculation of the relative integral area for the resonance at δ_iso_ = 3.20 ppm (attributed to CH_2_ groups in the polycarboxylate superplasticizer) for the ^1^H NMR data for each sample enables comparison of the relative amounts of the adsorbed superplasticizer in each case. Comparing these results (Figure 6) with the total organic carbon measurements (Figure 3A) shows the same overall trend, with addition of Ca^2+^ resulting in increased adsorption and addition of OH^–^ to raise the pH to 12.3 resulting in extensive desorption of the superplasticizer from silica fume.

Strong agreement between the experimental data obtained via total organic carbon and quantitative ^1^H NMR measurements was observed. The magnitude of the measured adsorption/desorption in each case shows a high degree of correlation. It has been shown for polycarboxylate ether (PCE) superplasticizers that the critical micelle concentration (CMC) is approximately 3 g/L in simulated cement pore solutions.^26^ This shows that micelle formation (to at least some extent) is likely to have occurred in each of the colloidal dispersions (and therefore the aqueous component obtained after separation from the solid component) and will result in low proton mobility and broad resonances, which may be unobservable in the ^1^H NMR data. This suggests that the very small differences in the magnitude of measured adsorption/desorption in Figure 6 are likely to be a result of the measurement technique used and rather than actual differences in adsorption/desorption.

**Figure 7:**
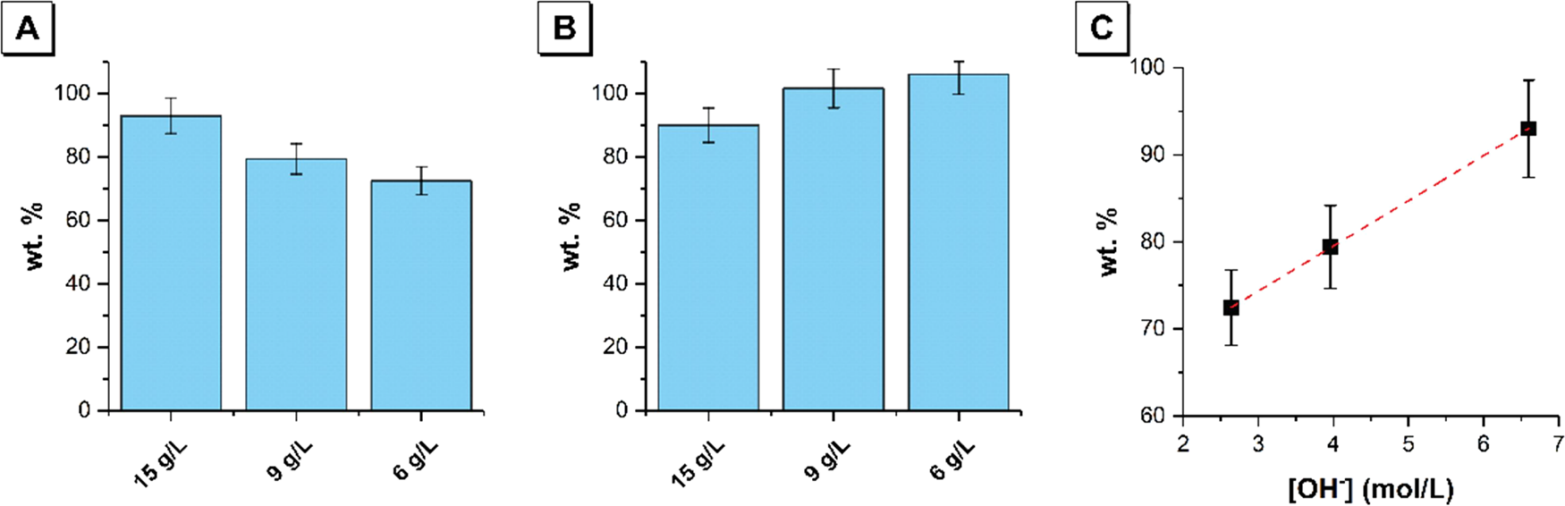
Comparison of the mass of total organic carbon remaining in the polycarboxylate solution after addition of 5 wt % silica fume and adjustment of pH by addition of OH^–^ (P + S + OH^–^), with (A) a concentration of NaOH of 440 mmol/g polycarboxylate in each solution and (B) a pH of 12.3 in each solution. Data are shown for polycarboxylate concentrations of 15, 9, and 6 g/L as marked. The relationship between the hydroxide ion concentration and the amount of polycarboxylate-based superplasticizer remaining in solution after filtration is shown in panel (C). The experiments were performed with a fixed concentration of NaOH of 440 mmol per gram of polycarboxylate in each solution and varying concentrations of the polycarboxylate superplasticizer (i.e., 15, 9, and 6 g/L). Consequently, the 15 g/L polycarboxylate solution will contain a higher concentration of NaOH than the 9 g/L polycarboxylate solution, which will in turn contain a higher concentration of NaOH than the 6 g/L polycarboxylate solution. In plot (C), the data points therefore represent the OH^–^ concentration in each polycarboxylate solution (e.g., for the 15 g/L polycarboxylate solution, [OH^–^] = 440 mmoL/g SP × 15 g SP/L H_2_O = 6.6 mol OH^–^ per L H_2_O). So, in plot (C), from left to right, the data points represent the OH^–^ concentration in the 6, 9, and 15 g/L polycarboxylate solutions.

Comparison of the mass of total organic carbon remaining in the polycarboxylate solution after addition of 5 wt % silica fume and adjustment of pH by addition of OH^–^ (P + S + OH^–^), with a fixed concentration of NaOH of 440 mmol/g polycarboxylate in each solution and varying concentrations of the polycarboxylate superplasticizer, is shown in Figure 7. This allows quantification of the effect of addition of hydroxyl ions on the sorption/desorption phenomena in these colloidal dispersions. The data show a greater amount of polycarboxylate superplasticizer remaining in solution after filtration (and hence less adsorption to the surface of silica) with increasing polycarboxylate concentration, even when the concentration of OH^–^ ions present relative to the mass of the polycarboxylate superplasticizer is held constant. When the pH is held constant, lower amounts of the polycarboxylate superplasticizer remain in solution after filtration with increasing polycarboxylate concentration. This is attributed to the change in anionic charge density on the polycarboxylate-based superplasticizer with adjustment of the concentration of OH^–^ ions^27^ due to deprotonation of the carboxylate groups in the polymer backbone.^28^ Increased anionic charge density results in increased electrostatic repulsion and hence desorption of the polycarboxylate from the silica surface.

### 3.2 Mechanism of Adsorption/Desorption of Polycarboxylate on Silica Fume

**Figure 8:**
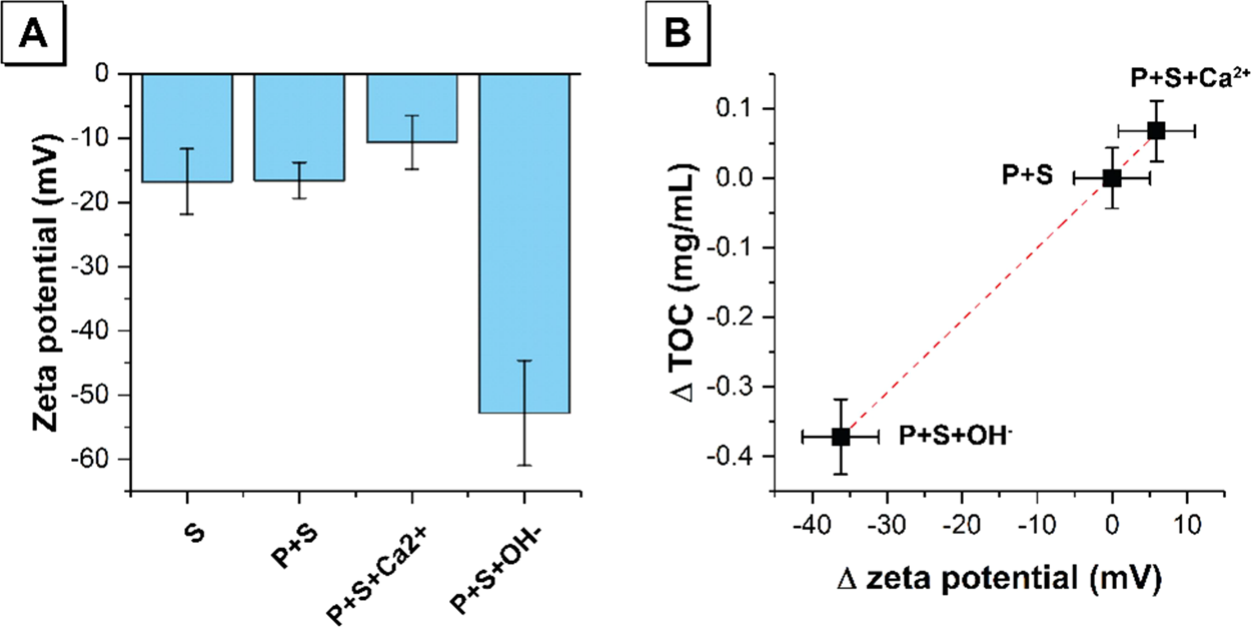
(A) Zeta potential of colloids formed after addition to the polycarboxylate solution of 5 wt % silica fume (P + S), addition of 5 wt % silica fume and adjustment of ionic strength by addition of Ca^2+^ (P + S + Ca^2+^), and addition of 5 wt % silica fume and adjustment of pH by addition of OH^–^ (P + S + OH^–^). Data are shown for a polycarboxylate concentration of 9 g/L. Zeta potential of silica fume in water (S) is shown for comparison. (B) Relationship between the difference in zeta potential of the colloid and mass of polycarboxylate desorbed from silica fume after adjustment of ionic strength by addition of Ca^2+^ (P + S + Ca^2+^) and adjustment of pH by addition of OH^–^ (P + S + OH^–^).

Zeta potential measurements shown in Figure 8A show that, at neutral pH, silica fume possesses a negative surface charge due to deprotonation of silanol groups when in solution.^6^ Addition of divalent cations (Ca^2+^) results in negative zeta potential of lower magnitude on the surface of the silica fume particles due to the adsorption of Ca^2+^ ions on the silica surface. This will drive further adsorption of the negatively charged polycarboxylate superplasticizer onto the silica surface via the electrostatic interaction with the positively charged Ca^2+^ ions. This adsorption mechanism is broadly analogous with previous works from Heinz et al.^29,30^ who used molecular dynamics simulations to show that the binding mechanism of PCEs containing ionic side groups on the surface of calcium silicate hydrate involves the initial migration of Ca^2+^ ions (present in the ionic side groups of the PCE) to the C–S–H surface followed by the anionic polymer backbone and subsequent conformation adjustments on the surface (e.g., tilted and partially upright conformations) resulting from local detachment from the surface due to electrostatic repulsion and steric effects arising from the common occurrence of more than two successive carboxylate groups in the PCE backbone. In the work presented here, adsorption is also promoted by a reduction of anionic charge density of the polycarboxylate-based superplasticizer due to complexation with Ca^2+^ ions and counter-ion condensation.^27,28^

Addition of OH^–^ ions results in a negative zeta potential of greater magnitude on the surface of the silica fume particles due to further deprotonation of silanol groups.^6^ The magnitude of the change in zeta potential after addition of OH^–^ ions is ∼6 times that after addition of Ca^2+^ ions. This indicates that, when both Ca^2+^ and OH^–^ ions are present (as occurs in the concrete mixes being simulated here, upon addition of the CaO-rich additive during mixing), an electric double layer will form on the surface of silica, with negatively charged hydroxyl groups forming on the surface of the adsorbed Ca^2+^ ions. This will result in a negative zeta potential on the surface of the silica fume particles, which will drive extensive desorption of the negatively charged polycarboxylate superplasticizer.^12^ Desorption is also promoted by an increase in the anionic charge density of the polycarboxylate-based superplasticizer due to deprotonation of the carboxylate groups in the polymer backbone^28^ and complexation with Ca^2+^ ions and counter-ion condensation.^27^ From the data obtained, there is a linear relationship between the change in zeta potential resulting from changes in aqueous chemistry and the amount of superplasticizer adsorbed to the silica fume surface (Figure 8B).

**Figure 9:**
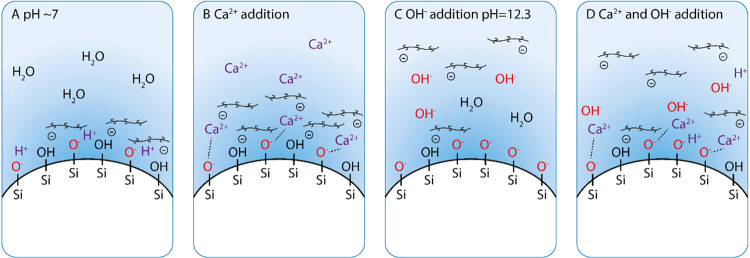
Schematic of surface charges on the silica surface at (A) pH 7 and after adjustment of (B) ionic strength by addition of Ca^2+^ (P + S + Ca^2+^), (C) adjustment of pH by addition of OH^–^ (P + S + OH^–^), and (D) adjustment of both ionic strength and pH by addition of Ca^2+^and OH^–^ ions. It should be noted that the polycarboxylate-based superplasticizer possesses a single negative valence in panel (B); this is purely for demonstrative purposes, and the charge on the polycarboxylate-based superplasticizer was not directly measured.

Together, the data obtained from the adsorption tests shows that dissolution of the CaO-rich additive in the cementitious mixture provides Ca^2+^ ions that shift the zeta potential to negative values of lower magnitude via adsorption of Ca^2+^ ions on the silica surface, and OH^–^ ions shift the zeta potential to negative values of greater magnitude via formation of an electric double layer and further deprotonation of surface silanol groups. Due to the greater magnitude of the shift in zeta potential due to OH^–^ ions, the net effect is a significantly greater negative charge on the silica surface. This mechanism is shown in Figure 9. This results in extensive desorption of the superplasticizer from silica fume. In fresh UHPC formulations containing particles of silica fume, blast furnace slag, and fly ash, this will allow a more even distribution of the superplasticizer across the inorganic SCM particles, allowing adsorption and dispersion of the solid particles. These findings provide an explanation for the rapid and extensive increase in fluidization with increasing Ca additive dose in fresh alkaline-earth-activated concretes,^13^ as shown in Figure 1.

The mechanism presented above extends previous works that demonstrated Ca^2+^-mediated adsorption of anionic polycarboxylate ether graft polymers (particularly allylether-based polycarboxylates) onto silica fume in alkaline media (pH = 12.8),^6,16^ with the findings presented here showing that the reversibility of polycarboxylate adsorption on silica fume is controlled by pH and results from the significantly greater effect of addition of OH^–^ ions on the magnitude of the zeta potential on the silica surface, compared with addition of Ca^2+^ ions. This is particularly important when using allylether-based polycarboxylate superplasticizers as these show strong preference for adsorption to silica fume over other inorganic cement or SCM particles^16^ (due to the carboxylate groups aligning more closely with the steric position of calcium ions situated at the surfaces of silica compared with cement or SCM particles). To counter this effect and allow adsorption to and dispersion of all solid particles in fresh UHPC, it has been suggested that a mixture of methacrylic acid ester-based polycarboxylate and allylether-based polycarboxylate superplasticizers should be used.^11^ In this work, we have presented a new mechanism that shows that, in alkaline-earth-activated UHPC, which exhibits significantly higher fresh state pH (>13) than those based on Portland cement (pH = 11), the adsorption polycarboxylate superplasticizers are more evenly distributed across the inorganic SCM particles due to the high concentration of OH^–^ ions. Chemical modeling will in the future enable further elucidation and confirmation of the chemical interactions demonstrated in this work, with important theoretical and practical implications for the use of very high-performing flowable concretes with a greatly reduced water content.

## 4 Conclusions

This study presents a new mechanism showing the effect of changes in pH, ionic strength, and charge on the reversibility of adsorption of a polycarboxylate-based superplasticizer on silica fume. The systems investigated were chemically simplified analogues of low-CO_2_, ultrahigh-performance concretes that exhibit significantly higher fresh state pH than those based on Portland cement.

The adsorbed amounts of the polycarboxylate-based superplasticizer on silica fume were measured using the depletion method. The amount of non-adsorbed superplasticizer remaining in solution after each adsorption experiment was determined by analyzing the total organic carbon content of the solution. This was independently confirmed by quantification of solution state ^1^H and ^13^C NMR measurements for each colloidal dispersion and by Fourier transform infrared spectroscopy measurements for the solids after adsorption. ^29^Si NMR and solid state ^29^Si MAS NMR measurements of silica fume before and after adsorption confirmed that the interaction between the polycarboxylate-based superplasticizer and silica fume is due only to surface adsorption. The mechanism of sorption/desorption of the polycarboxylate-based superplasticizer on silica fume was determined using zeta potential measurements of each colloidal dispersion.

Zeta potential measurements show that, at neutral pH, silica fume possesses a negative surface charge due to deprotonation of the silanol group. Addition of divalent cations (Ca^2+^) results in adsorption of the polycarboxylate-based superplasticizer on silica fume via (i) adsorption of Ca^2+^ ions on the silica surface and a negative zeta potential of lower magnitude on the surface of the silica fume particles and (ii) a reduction of anionic charge density of the polycarboxylate-based superplasticizer due to complexation with Ca^2+^ ions and counter-ion condensation. Addition of OH^–^ ions results in polycarboxylate desorption via deprotonation of silanol groups and a negative zeta potential of greater magnitude on the surface of the silica fume particles. The magnitude of the change in zeta potential after addition of OH^–^ ions is ∼6 times that after addition of Ca^2+^ ions.

Simultaneous addition of both Ca^2+^ and OH^–^ ions results in extensive desorption of the negatively charged polycarboxylate superplasticizer via (i) formation of an electric double layer on the surface of silica and hence a negative zeta potential on the surface of the silica fume particles and (ii) an increase in the anionic charge density of the polycarboxylate-based superplasticizer due to deprotonation of the carboxylate groups in the polymer backbone, complexation with Ca^2+^ ions, and counter-ion condensation. A linear relationship between the change in zeta potential resulting from changes in aqueous chemistry and the amount of superplasticizer adsorbed to the silica fume surface is observed.

These findings provide an explanation for the remarkable fluidizing effect that is observed upon addition of a very small amount (1.0 wt %) of a solid, powdered Ca source to fresh, low-CO_2_, ultrahigh-performance concretes that exhibit significantly higher fresh state pH (>13) than those based on Portland cement (pH 11).

## Supporting Information Available

Video showing the effect of addition of the powdered Ca source during mixing of a high-strength alkaline-earth-activated concrete (MP4)
